# How calcite and modified hydroxyapatite influence physicochemical properties and cytocompatibility of alpha-TCP based bone cements

**DOI:** 10.1007/s10856-017-5934-3

**Published:** 2017-07-05

**Authors:** Aneta Zima, Joanna Czechowska, Dominika Siek, Radosław Olkowski, Magdalena Noga, Małgorzata Lewandowska-Szumieł, Anna Ślósarczyk

**Affiliations:** 1 Faculty of Material Science and Ceramics, AGH University of Science and Technology, 30 Mickiewicza Av., 30-059 Krakow, Poland; 2 Department of Histology and Embryology, Center for Biostructure Research, Medical University of Warsaw, 5 Chałubińskiego St., 02-004 Warsaw, Poland; 3 Department of Pathology, Medical University of Warsaw, 5 Chałubińskiego St., 02-004 Warsaw, Poland; 4 Centre for Preclinical Research and Technology, Medical University of Warsaw, 1B Banacha St., 02-097 Warsaw, Poland

## Abstract

**Abstract:**

Nowadays successful regeneration of damaged bone tissue is a major problem of the reconstructive medicine and tissue engineering. Recently a great deal of attention has been focused on calcium phosphate cements (CPCs) as the effective bone fillers. Despite a number of studies regarding CPCs, only a few compare the physicochemical and biological properties of α-TCP based materials of various phase compositions. In our study we compared the effect of several components (calcite, hydroxyapatite doped with Mg^2+^, CO_3_^2−^ or Ag^+^ ions, alginate, chitosan and methylcellulose) on the physicochemical and biological properties of α-TCP-based bone cements. The influence of materials composition on their setting times, microstructure and biochemical stability in simulated body fluid was determined. A number of in vitro laboratory methods, including ICP-OES, metabolic activity test, time-lapse microscopic observation and SEM observations were performed in order to assess biocompatibility of the studied biomaterials. The positive outcome of XTT tests for ceramic extracts demonstrated that all investigated cement-type composites may be considered cytocompatible according to ISO 10993-5 standard. Results of our research indicate that multiphase cements containing MgCHA, AgHA and calcite combined with αTCP enhanced cell viability in comparison to material based only on αTCP. Furthermore materials containing chitosan and methylcellulose possessed higher cytocompatibility than those with alginate.

**Graphical abstract:**

## Introduction

Due to the increasing need for bone defects replacement, a number of intensive studies are performed to find perfect bone substitutes, which would not only mimic the chemical and mechanical properties of the natural bone but also stimulate its regeneration. Calcium phosphate bone cements (CPCs) are of particular interest mainly due to their osteoconductive properties and excellent handling. CPCs are defined as blends of calcium phosphates powders, which when mixed with an aqueous solution form a viscous paste that sets *in situ*. The moldable form of material allows for the optimal adaptation to the shape of bone defect [1, 2]. Calcium phosphate cements have been widely used in medicine due to their biocompatibility, bioactivity, easy molding, direct contact with bone tissue since the first stage of implantation as well as setting without highly exothermic reaction, which prevents necrosis of surrounding tissue [3]. The CPCs compositions have been constantly evaluated and improved. The major challenges for the future are transforming the present biocements into fully degradable materials and improving their mechanical strength and toughness [4]. Development of ion substituted calcium phosphates used as substrates offers the potential to adjust mechanical properties and bone remodeling of bone cements in vertebroplasty [5].

In the recent years a great attention has been focused on α-TCP based bone cements, which have been extensively investigated in order to optimize their physicochemical and biological properties [6, 7]. It has been proven that the properties of synthesized αTCP powder depend on the applied substrate, the way of synthesis as well as the synthesis conditions. Cicek et al. have found the correlation between setting process pathway of α-TCP and the amount of impurities in CaCO_3_ precursor [8]. The effect of morphology, crystallinity and particle size of α-TCP on the kinetics of setting reaction was also reported [9]. Besides the way of producing α-TCP powder the physicochemical and biological properties of set and hardened cement bodies depend also on the powder batches composition as well as the liquid phase type [10]. In order to optimize setting times, mechanical properties as well as bioresorbablility and biocompatibility of α-tricalcium phosphate based materials a number of inorganic additives including dicalcium phosphate (CaHPO_4_, CaHPO_4_·2H_2_O), calcium carbonate (CaCO_3_) and calcium sulphate (CaSO_4_·1/2H_2_O, CaSO_4_·2H_2_O) have been used [11]. Combes et al. [12] revealed excellent biocompatibility of CaCO_3_–CaP cements and stated that the presence of controlled amounts of CaCO_3_ might increase the resorption of composite and favors its replacement by bone tissue. Due to the great potential of hydroxyapatite to host a variety of ions in its crystal lattice, ion substitution of HA has become the subject of intense investigations. The synergistic effect of Mg and CO_3_ on reducing crystallinity and increasing the extent of dissolution has been confirmed in the previous studies [13]. Besides a biomimetic approach, introduction of antibacterial agents into the cement structure, especially silver is the subject of intensive research. Silver doped hydroxyapatite may serve as the antibacterial constituent of biocomposites [14]. The combination of hydrogels and calcium phosphates is emerging trend for bone grafts. It is believed that use of polymers such as alginate, chitosan, cellulose and their derivatives allows improve cohesion, injectability and mechanical strength of CPCs. Furthermore composites with excellent biocompatibility may be obtained by admixing those organic constituents with calcium phosphates [15, 16]. Cytocompatibility of material is the most important factor in determining its usability as a future tissue engineering product. Behavior of osteoblasts on artificial surfaces is dependent on the cell culture conditions [17]. There are a number of parameters that influence the expression of the osteoblastic phenotype including the type of culture medium, number of passages and the presence of compounds [18]. The clinical potential of biomaterials may be evaluated in vitro using complex aqueous solutions, such as simulated body fluid or tissue culture medium, often in combination with cells and proteins. These simple model systems allow for an initial screening of possible reactions induced by the material. Cell dynamics can be efficiently investigated in vitro via live cell imaging by time lapse microscopy (TLM). Visualization of biological systems during their dynamic evolution is possible [19]. The application of image analysis techniques allows for examining cell trajectories and migration parameters or for the quantification of the growth dynamic of tissues [20, 21]. The cellular response to CPCs is linked to their phase composition [22]. The effect of chemical stability and resorbability of biomaterials, including ion release, on the cellular response was confirmed inter alia by Malafaya and Reis [23] and Przekora et al. [10].

The aim of the present work was to carry out a systematic study how calcite and modified hydroxyapatite influence physicochemical properties and cytocompatibility of α-TCP based bone cements. α-TCP was synthesized in the alternative way, i.e., by using a wet chemical method [24]. As the components of solid phases: calcite or HA modified with Ag^+^, Mg^2+^, CO_3_^2−^ ions were applied. In order to optimize setting times and enhanced cellular response of cement-type composites various liquid phases, namely: sodium alginate, chitosan and methylcellulose solutions were used. The method of parallel evaluation of ionic release and cytocompatibility of materials was proposed.

## Materials and methods

### Materials

The initial α-TCP powder was synthesized by the wet chemical method using Ca(OH)_2_ (POCH, Poland) and H_3_PO_4_ (POCH, Poland) as reactants. During the synthesis pH ranged from 5.0 to 5.5. The obtained precipitate was dried, sintered above 1250 °C (5 h) and ground in an attritor mill in the presence of ethyl alcohol (3 h). Thus obtained α-TCP powder was sieved below 0.063 mm. Hydroxyapatite powders were synthesized using the wet chemical methods. CaO (POCH, Poland), H_3_PO_4_ (POCH, Poland) and CH_3_COOAg (Chempur, Poland) were applied as the sources of calcium, phosphorus and silver ions, respectively in silver doped hydroxyapatite (AgHA). Hydroxyapatite doped with Mg^2+^ and CO_3_^2−^ ions (MgCHA) was synthesized using CaO, (NH_4_)_2_HPO_4_, (CH_3_COO)_2_Mg and NH_4_HCO_3_ as reagents. During synthesis, the pH value of the mixtures was stabilized at 11 using ammonium hydroxide solution. The suspension was aged for 24 h at room temperature and then decanted. The precipitate was washed with distilled water, filtered and dried at 90 °C. Hydroxyapatite powders were ground in a ball mill and sieved below 0.063 mm.

Three types of initial powder batches composed of monophasic α-TCP or α-TCP mixed with calcite (POCH, Poland), AgHA or MgCHA at the 3:2 weight ratio were produced using the ball mill (MM 400 Retsch). Cement samples were prepared by mixing initial powder batches with liquid phases: sodium alginate, chitosan and methylcellulose solutions (Table 1). Medium molecular weight chitosan (around 100,000 kDa) and methylcellulose were purchased from Sigma-Aldrich, whereas sodium alginate from Acros Organics.

**Table 1:** The initial composition of powder batches and liquid phases of the studied materials

Material	Solid phase	Liquid phase
T-1	α-TCP	1.0 wt% sodium alginate in 2.0 wt% Na_2_HPO_4_
TC-1	α-TCP: calcite (3:2)	1.0 wt.% sodium alginate in 2.0 wt% Na_2_HPO_4_
TC-2	α-TCP: calcite (3:2)	1.0 wt% chitosan in 2.7% acetic acid
T-AgHA	α-TCP: AgHA (3:2)	0.75 wt% methylcellulose in 2.0wt.% Na_2_HPO_4_
T-MgCHA	α-TCP: MgCHA (3:2)	0.75 wt% methylcellulose in 2.0 wt% Na_2_HPO_4_

### Methods

#### Physicochemical characterization

##### Phase composition

The crystalline phases of the set and hardened cement bodies were analyzed by powder X-ray diffraction with CuK_α_ radiation (D2 Phaser, Bruker) within the 2θ range from 10° to 60° at a scanning speed of 1°/min. The phases were identified by comparing the experimental X-ray diffractograms to the standards compiled by the Joint Committee on Powder Diffraction Standards (JCPDS): HA (JCPDS 01-070-0798), α-TCP(JCPDS 00-009-0348) and calcite (JCPDS 00-003-0569). Each measurement was repeated three times. Phase quantification was calculated using the Rietveld method. Specific surface areas of initial powders (α-TCP, AgHA, MgCHA, and calcite) were determined by BET method (ASAP 2010 Micromeritics).

##### Setting times

Setting times of the cement pastes were determined using Gilmore Apparatus according to the ASTM C266-08 standard [25]. The Gillmore Apparatus possess two needles: the initial setting time needle with a tip diameter of 2.12 ± 0.05 mm and a mass of 113.0 ± 0.5 g and the final setting time needle with a tip diameter of 1.06 ± 0.05 mm and a mass of 453.6 ± 0.5 g. To measure the setting time the needles were lightly applied to the surface of the paste. The setting time end point is a penetration measurement that does not mark the cement surface with a complete circular sign. The cement samples (8 × 10 × 5 mm) were prepared by mixing powder batches with the liquid phases at given L/P ratios. The powder to liquid ratio was chosen in terms of equal workability of appropriate consistency for each cement composition. All experiments were performed at room temperature (23 ± 2 °C). Each measurement was repeated six times and an average value was calculated.

##### Open porosity

The open porosity and pore size distribution were studied by mercury porosimeter (AutoPore IV 9500 Porosimeter, Micromeritics). Cement samples were dried and afterward introduced into the penetrometer, which was subsequently placed in the low- and high- pressure chamber of the apparatus. Mercury penetration volume was registered while increasing the intrusion pressure. Open porosity and pore size distributions were generated from the pressure vs. intrusion data using the Washburn equation [26].

##### Biochemical stability

Biochemical stability was evaluated using inductively coupled plasma optical emission spectrometry (ICP-OES). The samples 12 ± 1 mm of diameter and 4 ± 1 mm of high and 0.65 ± 0.15 g of weight were incubated in 40 ml of SBF at 37°C. Changes in the concentration of some substantial chemical elements, namely: Ca, Na, K, Mg, P and Ag in SBF after 1, 3 and 7 days of samples incubation. Measurements were performed using Perkin-Elmer spectrometer Optima 2100. ICP-OES instrumental conditions are collected in Table 2. Their correctness was verified by analyzing the “Groundwater, high” (CRM ES-H-2, SCP Science, Canada) as the reference material. Each measurement was repeated three times. Results were presented as mean value ± standard deviation.

**Table 2:** ICP-OES instrumental conditions

Plasma	Argon
Nebulizer gas flow rate	0.8 l min^−1^
Auxiliary gas flow rate	0.2 l min^−1^
Plasma gas flow rate	15.0 l min^−1^
RF Generator power	1300 W
Flow rate of sample	1.5 ml min^−1^
Plasma observation axial	(Mg, Ca P I S), radial (Na, K)
Reading parameters peak area	Five points per peak
Measurements	Three replicates, reading time—auto

#### Evaluation of biocompatibility

##### Cell line

In order to evaluate biocompatibility of investigated materials the osteoblast-like human osteosarcoma cell line MG-63 (CRL-1427, ATCC, Manassas, VA, USA) has been used for in vitro tests. The cells were grown in standard culture medium (Dulbecco’s modified Eagle medium, 10% Fetal Bovine Serum, 1% Penicillin-Streptomycin and 2 mM l-glutamine, Life Technologies). At subconfluency cells were detached using trypsin solution (Life Technologies), centrifuged and counted in hemocytometer. An appropriate cell number was seeded into the culture plate wells or onto the ceramic samples.

##### Cytotoxicity of ceramic extracts

MG-63 cells were seeded at a density of 10,000 cells per well on 96-well plate. Ceramic extracts of all investigated samples were prepared in accordance with ISO 10993-12 standard [27] and biocompatibility of cements was analyzed according to 10993-5 standard [28]. Samples with culture medium (0.1 g ceramic sample per 1 ml culture medium) were incubated for 24 h at 37 °C. Afterwards extracts were centrifuged (5000 rpm, 10 min) to remove all ceramic debris. Then cell-populated wells of 96-well plate were filled with 100 μl of each extract or 100 μl of standard medium in control wells. The cells were cultured for 24 h. Afterwards the morphology of cells was examined with an inverted microscope (Nikon Eclipse TE2000-U, Kanagawa, Japan). Metabolic activity of MG-63 cells cultured with extracts was determined with XTT assay (Sigma-Aldrich, St. Louis, MO, USA) [29]. Absorbance was measured at microplate reader at the wavelength of 450 nm (Fluostar Optima, BMG Labtech, Germany).

#### Observation of cells in direct contact with materials

##### Time-Lapse experiment

MG-63 cells were plated at a density of 12,000 cells per well on 48-well plate. After 24 h of culture, medium was removed and ceramic samples were placed into the cell-seeded wells of culture plate with 900 μl of medium, each investigated sample in the separate well. Cells were cultured in the contact with materials in the special chamber for real time observation (37 °C, 5% CO_2_, 95% humidity) and observed under inverted microscope over 3 days (Nikon Eclipse TE2000-E, Kanagawa, Japan). The pictures of proliferating cells in culture with α-TCP-based biocements were taken every 10 min during the time of culture.

##### SEM observations

In order to analyse impact of α-TCP-based cements on cells cultured directly on their surface, cell morphology was analyzed by scanning electron microscopy. Samples of cements were pre-incubated for 1 h in culture medium, then medium was removed and samples were placed into 24-well culture plate. 25 μl of cell suspension with 20 000 MG-63 cells was pipetted onto each sample surface, cells were allowed to adhere for 1 h, then volume of culture medium was filled up to 1 ml and in vitro culture was continued for 48 h. Afterwards samples were washed with PBS (Life Technologies), fixed and dehydrated. Observation was done in TM3000 (Hitachi High-Technologies, Japan) scanning electron microscope.

### Statistical analysis

The results of cell viability assays were presented as mean ± SD. To evaluate the significance of the differences Kruskal–Wallis non-parametric ANOVA with following post hoc test was performed using STATISTICA 10 (StatSoft, Tulsa, OK, USA). For statistical analyses, *P* ˂ 0.05 was considered statistically significant.

## Results

### Physicochemical characteristics

#### Phase composition

**Fig. 1:**
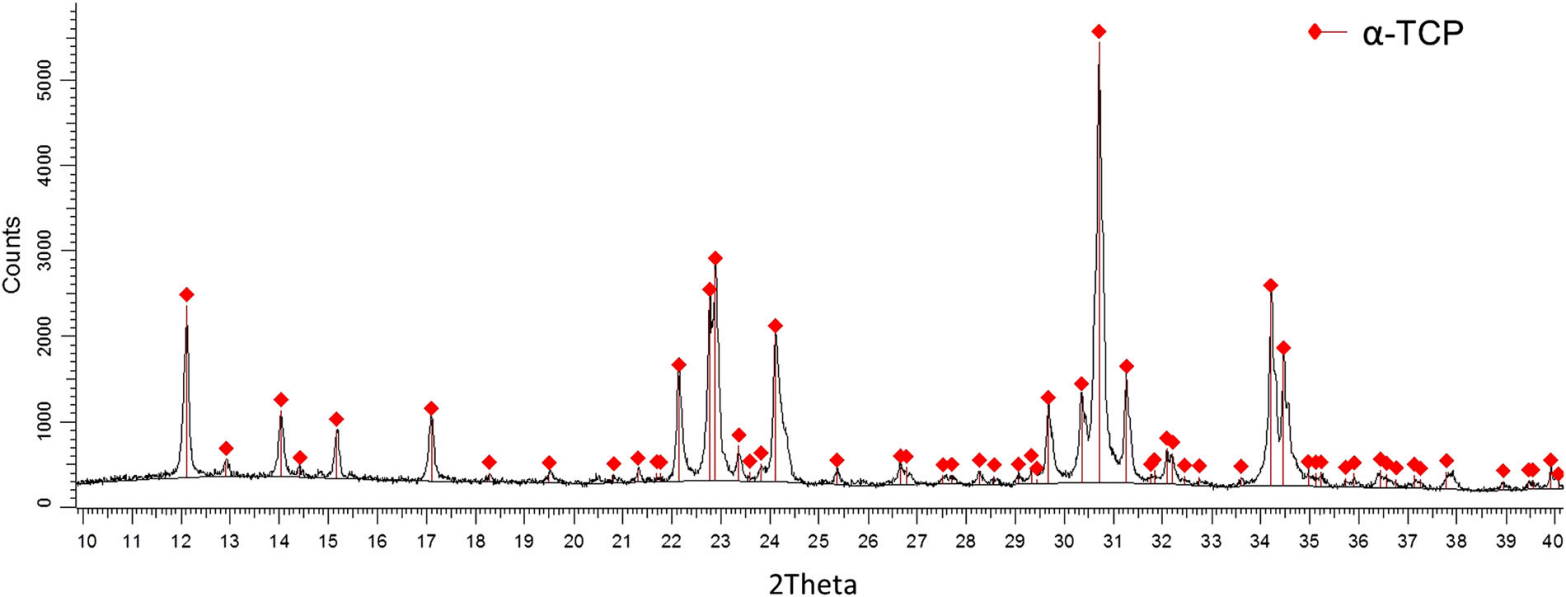
X-ray diffractogram of the initial α-TCP powder synthesized via the wet chemical method

Results of XRD (Fig. 1) and BET studies revealed that the initial α-TCP was a monophasic powder with specific surface area (SSA) equal 5.28 ± 0.02 m^2^/g. The values of SSA of initial powders (αTCP, AgHA, MgHA and calcite) are presented in Table 3. XRD measurements demonstrated that 7 days after setting hardened cement bodies composed of α-TCP and hydroxyapatite (T1, T-AgHA and T-MgCHA) or α-TCP, HA and calcite (TC-1 and TC-2) (Table 4). In the case of materials T-AgHA and T-MgCHA the process of α-TCP hydrolysis to calcium deficient hydroxyapatite was faster in comparison with composites consisting of α-TCP and calcite. 7 days after setting and hardening higher amount of hydroxyapatite was observed for material T-AgHA (78.0 wt%) than for T-MgCHA (67.8 wt%). In the case of materials T-1, T-AgHA and T-MgCHA, after 7 days of incubation in SBF of cement samples, α-TCP almost completely hydrolyzed to hydroxyapatite (from 94.9 to 97.4wt%). For cements containing calcite inhibition of α-TCP hydrolysis to HA was observed.

**Table 3:** Specific surface area and phase composition of the initial powders

Initial powder	Specific surface area (m^2^/g)	Phase composition		
		α-TCP	HA	Calcite
αTCP	5.28 ± 0.02	93.4 ± 2.0	6.6 ± 2.0	–
AgHA	25.20 ± 0.02	–	99.5 ± 0.5	–
MgCHA	99.80 ± 0.20	–	100.0 ± 0.0	–
Calcite	0.24 ± 0.01	–	–	100.0 ± 0.0

**Table 4:** Phase composition of the studied materials 7 days after setting and hardening before and after incubation in SBF

Symbol of material	Phase composition					
	Non-incubated in SBF			Incubated in SBF		
	α-TCP (wt%)	HA (wt%)	Calcite (wt%)	α-TCP (wt%)	HA (wt%)	Calcite (wt%)
T-1	75.8 ± 4.8	24.0 ± 4.9	–	5.1 ± 1.7	94.9 ± 1.6	–
TC-1	40.0 ± 1.8	21.5 ± 2.8	38.6 ± 1.1	17.1 ± 0.6	45.8 ± 2.7	37.1 ± 2.1
TC-2	44.4 ± 1.4	16.3 ± 2.3	39.3 ± 1.0	22.6 ± 1.0	37.1 ± 2.1	40.4 ± 1.1
T-AgHA	22.0 ± 3.4	76.8 ± 3.4	–	2.6 ± 1.0	97.4 ± 2.7	–
T-MgCHA	30.06 ± 2.4	69.4 ± 2.4	–	4.2 ± 2.4	95.8 ± 2.4	–

#### Setting times

Setting times of the developed materials varied with their composition and ranged from 6 to 17 min-initial and from 13 to 35 min-final (Table 5). The lowest liquid to powder ratio (0.32–0.34 g/g) and the longest setting times were observed for cements containing calcite (TC-1, TC-2). Materials T-AgHA and T-MgCHA, containing AgHA and MgCHA, respectively required more liquid phase to obtain the appropriate consistency of cement paste than the rest of examined CPCs. It is probably connected with high specific surface area of MgCHA (99.8 ± 0.2 m^2^/g) and AgHA (25.2 ± 0.02 m^2^/g) powders. Furthermore, those cements possessed slightly longer final setting times than material produced solely of α-TCP without inorganic additives (T-1). Polymers introduced into the material with the liquid phase played the role of cohesion promoter, providing the required viscosity and consistency of the cement pastes.

**Table 5:** Initial and final setting time of the cement pastes

Material	L/P (g/g)	Setting time (min)	
		Initial (I)	Final (F)
T-1	0.48	7 ± 1	13 ± 1
TC-1	0.34	11 ± 1	21 ± 1
TC-2	0.32	17 ± 1	35 ± 1
T-AgHA	0.60	6 ± 1	19 ± 1
T-MgCHA	0.56	6 ± 1	17 ± 1

#### Open porosity

**Fig. 2:**
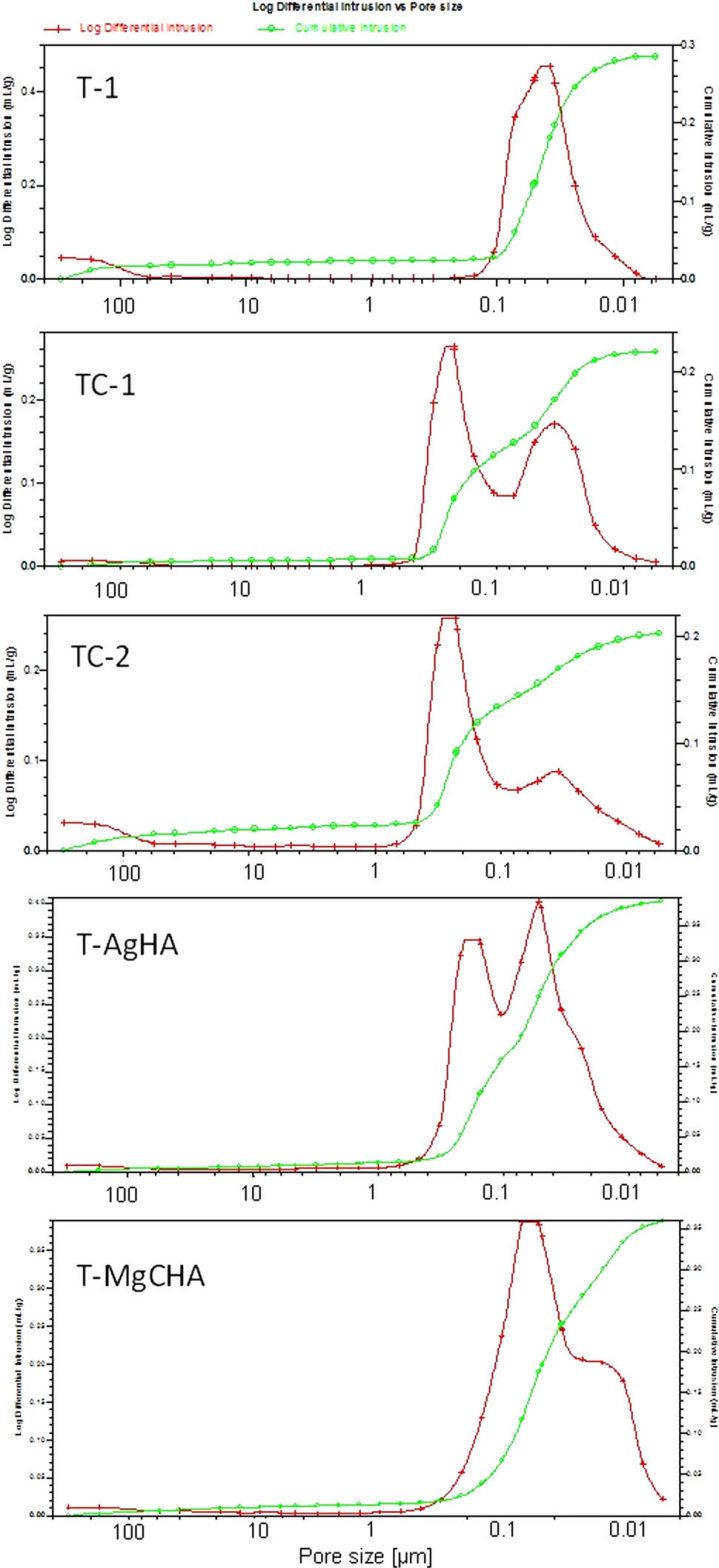
Pore size distribution of the investigated materials

The results of the mercury porosimetry measurements are presented in Fig. 2. Analysis of pore size distribution has been carried out in a wide pore size range, from 350 µm up to 0.005 µm, however hardened cement bodies showed porosity in a narrow range of pores 0.480–0.006 µm. A bimodal pore size distribution was observed for all materials except T-1. Open porosity of the obtained cements differed depending on the composition and ranged from 35 to 51 vol.%. The lowest porosity values, i.e., 35 and 39 vol.%, were noted for materials TC-1 and TC-2, respectively (containing calcite). Materials consisting of α-TCP and modified hydroxyapatite showed the open porosity ranging from 48 to 51 vol.%.

#### Biochemical stability

Changes in the concentrations of selected elements in SBF after different incubation periods of the developed materials are collected in Table 6. The results of ICP-OES measurements revealed a decrease in concentration of calcium as the function of time in the case of all materials, except for TC-2, in comparison to the control. This effect was particularly visible for material T-MgCHA. A substantial decrease in Mg concentration in the case of cement T-1 was noticed, whereas an increase in the amount of magnesium was seen for TC-2. The concentration of magnesium for the rest of materials was at the level of control. Continues release of small amounts of silver from the material T-AgHA, consisting of α-TCP and Ag-doped hydroxyapatite, was observed.

**Table 6:** Concentrations of elements in simulated body fluid after different periods of incubation of the cement samples material

Material	Time (day)	Concentration (mg/l)					
		Ca	K	Mg	Na	P	Ag
Control (SBF)	1	88.87 ± 1.78	276.79 ± 5.54	26.52 ± 0.53	3753.90 ± 93.85	34.00 ± 0.68	–
T-1	1	72.57 ± 1.45	271.90 ± 8.16	5.57 ± 0.11	3930.4 ± 78.61	32.84 ± 0.66	–
	3	72.26 ± 1.81	268.08 ± 7.51	3.08 ± 0.08	3825.2 ± 95.63	32.49 ± 0.81	–
	7	56.71 ± 1.70	268.46 ± 6.71	3.29 ± 0.10	3932.1 ± 94.37	28.68 ± 0.86	–
TC-2	1	61.74 ± 1.23	197.11 ± 5.91	38.26 ± 0.77	2813.2 ± 56.26	21.10 ± 0.42	–
	3	93.14 ± 2.33	282.63 ± 7.35	37.04 ± 0.93	3945.8 ± 98.65	23.76 ± 0.59	–
	7	91.06 ± 1.91	281.94 ± 7.33	34.52 ± 0.72	3941.6 ± 82.77	18.75 ± 0.39	–
T-AgHA	1	77.77 ± 2.18	291.04 ± 8.73	29.04 ± 0.81	3783.99 ± 98.38	31.56 ± 0.88	0.43 ± 0.01
	3	75.53 ± 1.89	222.64 ± 6,23	29.10 ± 0.73	3104.92 ± 77.62	32.26 ± 0.81	0.71 ± 0.06
	7	53.25 ± 0.96	350.80 ± 8.07	21.41 ± 0.39	4615.14 ± 83.07	33.22 ± 0.60	1.31 ± 0.02
T-MgCHA	1	57.93 ± 1.10	229.68 ± 5.97	22.05 ± 0.42	3266.76 ± 62.07	30.57 ± 0.58	–
	3	53.38 ± 1.07	251.85 ± 7.05	19.58 ± 0.39	3467.56 ± 69.35	25.84 ± 0.52	–
	7	40.18 ± 1.21	286.91 ± 7.46	16.64 ± 0.50	3960.88 ± 79.22	20.97 ± 0.63	–

#### Biocompatibility

**Fig. 3:**
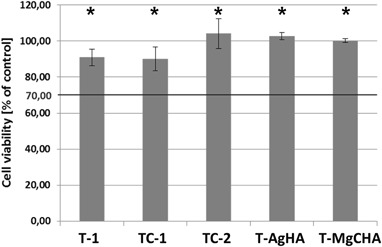
XTT assay results presented as a percentage of the cellular metabolic activity of MG-63 cells cultured in 100% ceramic extracts in comparison to the control (standard culture medium). For each extract cell viability is significantly higher than 70% of control viability, therefore each extract may be considered cytocompatible. *Asterisks* denote statistically significant difference (*P* < 0.05) between the samples and the value of 70% of control. However, there were no significant differences between the results obtained for the investigated materials

**Fig. 4:**
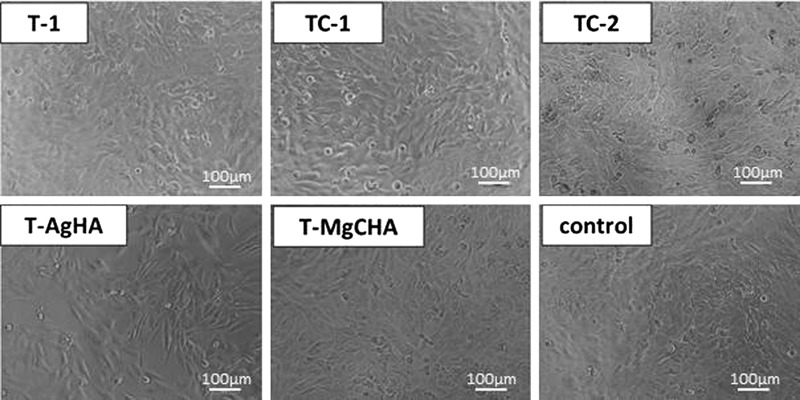
Morphology of MG-63 cells cultured in 100% extracts and standard culture medium (control) performed with inverted light microscope

In order to evaluate the viability of MG-63 cells in culture with 100% ceramic extracts the XTT assay was performed. The results of studies revealed that all investigated samples met requirements of ISO 10993-5 standard for cytocompatibility of materials, i.e., cells cultured in ceramic extracts possessed over 70% of viability of cells cultured in the standard medium. For two of tested materials: TC-2, T-AgHA, cells showed metabolic activity higher than the control (Fig. 3). Composites containing chitosan and methylcellulose possessed higher cytocompatibility (~100% of control) than those with alginate (~80% of control). The inverted light microscopy observation (Fig. 4) revealed comparable number of cells in four out of five investigated materials. A proper morphology of cells with normal elongated, spindle shape was observed for all the examined materials. Confluent cell culture, where all avaiable surfaces were covered by cells, was observed in the case of materials: T-1, TC-1, TC-2 and T-MgCHA, whereas for T-AgHA sample a subconfluency of culture was achieved.

#### Time-lapse microscopy observation

**Fig. 5:**
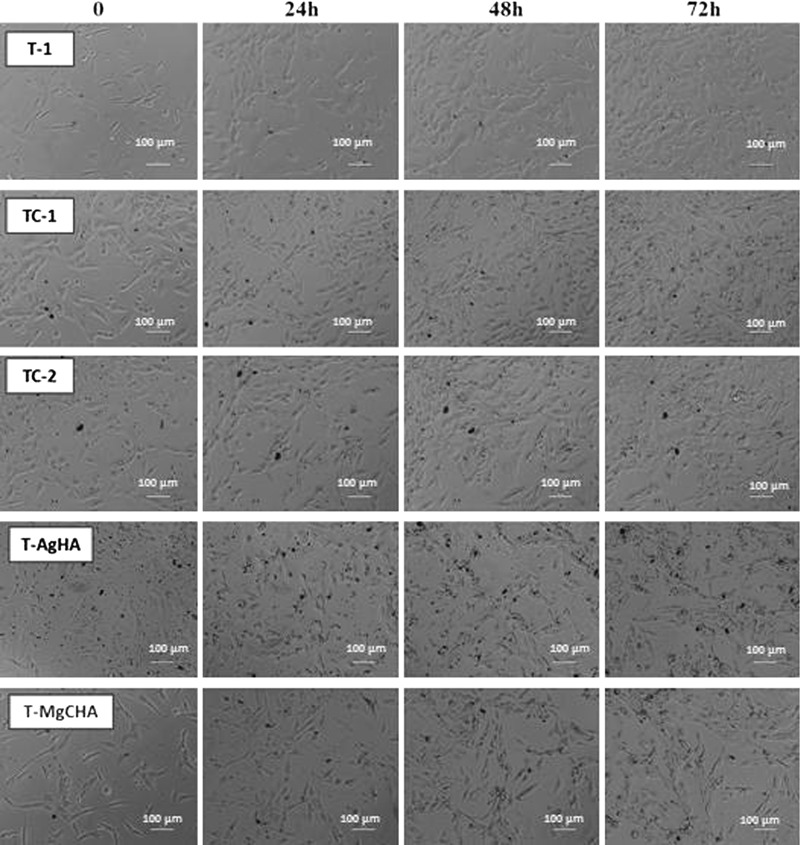
Morphology and proliferation changes of MG-63 cells cultured in close contact with investigated ceramic materials in 48-well plate. Samples were placed into wells seeded with MG-63 cells. Time-lapse pictures of MG63 cells in five investigated conditions presented at four selected time points

In order to estimate the influence of the obtained materials on osteoblast-like human osteosarcoma cells, both cell proliferation and morphology were investigated. During the microscopy observation no negative impact of investigated materials on cell proliferation or morphology was observed. Regular cell spreading on the culture surface as well as numerous mitosis were observed. As shown in Fig. 5, the number of cells increased with time. Some ceramic debris from the materials TC-1, TC-2, T-AgHA, T-MgCHA were present in the systems (black dots). Debris resulted from biocements disintegration occuring in vitro during the dissolving and degradation processess. Presence of debris did not disrupt the cell functions and proliferation.

#### SEM observations

**Fig. 6:**
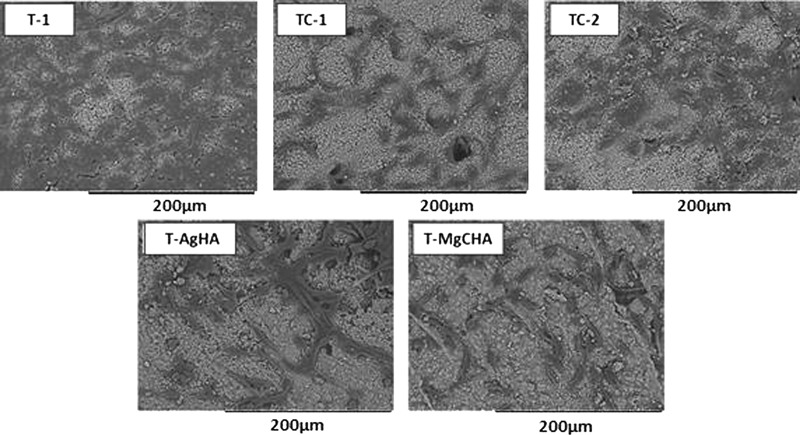
SEM observation of MG-63 cells on surface of the cement samples after 2 days of in vitro culture in 48-well plate

Early in vitro cytocompatibility studies focused on the morphological aspect, as well as growth capacity of cells on biomaterials. In our study after 2 days of cell culture on the materials in 24-well plate we found cells spread on the surface of all investigated samples and visualized them in SEM studies (Fig. 6). Numerous cells with proper morphology were observed. The greatest number of attached cells was visible on the material T-1 (α-TCP without the inorganic additive). In the case of the rest of examined biocements no significant differences in the number of cells were observed.

## Discussion

This study aims to examine the influence of inorganic and organic additives on physicochemical properties and cytocompatibility of apatite, α-TCP based cements. It is well known that α-TCP at near physiological temperature hydrolyzes to hydroxyapatite [30]. In this work, α-TCP was synthesized using a wet chemical method [24]. As compared to α-TCP produced via solid state reaction, differences in hydraulic properties, resorption rate and material-cell interaction were expected. According to the obtained results, a part of α-TCP present in the studied materials hydrolyzed to a calcium-deficient hydroxyapatite according to Eq. 1:

1 $$3 {\rm{\alpha }}-{\rm{C}}{{\rm{a}}_3}\,{\left( {{\rm{P}}{{\rm{O}}_4}} \right)_2}{\rm{ + }}{{\rm{H}}_{\rm{2}}}{\rm{O}} \to {\rm{C}}{{\rm{a}}_9}\ \left( {{\rm{HP}}{{\rm{O}}_4}} \right)\ {\left( {{\rm{P}}{{\rm{O}}_4}} \right)_5}\ \left( {{\rm{OH}}} \right)$$

Hydroxyapatite substituted with magnesium, carbonate or silver ions is expected to produce enhanced biological and chemical responses in the body and when applied in bone cements, it will influence their properties. Presence of calcite is supposed to increase the resorption rate of the composite. The presence of doped hydroxyapatite in the initial compositions of the studied materials (T-AgHA, T-MgCHA) accelerates creation of higher amount of non-stoichiometric hydroxyapatite, which is similar to the inorganic component of the bone tissue due to ionic substitutions in crystal structure. Setting time requirements proposed in the literature for CPCs are as follows [14]: about 4–8 min initial and about 10–15 min final. Most of calcium phosphate cements show longer setting times, which depend mostly on the initial powder batches and liquid phase composition. As expected, the liquid phase type influenced the setting process of the developed materials. When chitosan in acetic acid solution was applied (TC-2) noticeable longer setting time in comparison to the materials with other liquid phases, namely: sodium alginate in Na_2_HPO_4_ solution (TC-1, T-1) and methylcellulose in Na_2_HPO_4_ solution (T-AgHA, T-MgCHA) was observed (Table 4). These results are in accordance with previous studies conducted by Ginebra et al. [30] and Momota et al. [31], who reported that Na_2_HPO_4_ accelerates setting of apatitic calcium phosphate cements. Results of our tests confirm also the results obtained by Czechowska et al. [7] indicating that chitosan in acetic acid solution may serve as a retarder of α-TCP setting process. Intrinsic porosity is one of the most important features of CPCs. All obtained cements are microporous with the open porosity in the range of 35 to 51 vol.%. Materials consisted of α-TCP and modified hydroxyapatite possessed higher open porosity (48–51%) than those with α-TCP and α-TCP with calcite (35–42%). The observed increase in porosity was probably due to higher liquid to powder ratio (L/P) required in the case of materials T-AgHA and T-MgCHA in comparison to TC-1 and TC-2 ones. Increasing L/P ratio causes the formation of higher amount of larger pores due to the longer distance between the particles of setting phase. Similar effect of L/P ratio on cement porosity was observed by Espanol et al. [32] and Ginebra et al. [33].

The process of cell interaction with materials is highly dynamic and depends on various parameters influencing the cell responses [34, 35]. The final success of bone implant materials largely depends upon the formation of strong, mechanically stable interface between the material and bone tissue. Material composition as well as its surface chemistry, topography and energy play an essential part in osteoblast adhesion, organization and mineralization in vitro [34, 36]. The attachment, adhesion and spreading occur in the first stage of cell/material interaction. This first phase will influence the ability of cells to proliferate and differentiate in contact with the implants. Solution mediated surface reactions influence in vitro and in vivo behavior of implant materials. The differences in the concentration of chemical elements in the culture medium results from both, phase composition and microstructure of incubated materials. It is known that osteoblasts interact with the material’s surface already conditioned by the biological fluid components. The properties of materials and simulated biological fluids influence proteins adsorption and osteoblasts behavior. Ionic composition and pH of the solution as well as the functional group of proteins and substrates determine protein adsorption on the material surface [17, 34]. Furthermore, phenomena associated with ion release from implant materials may be crucial for cells viability in vitro and in vivo [37]. The results of previously conducted studies demonstrated that the critically low concentration of some ions, especially Ca^2+^, Mg^2+^ might inhibit the activation of integrins (transmembrane receptors—bridges for cell–cell and cell-extracellular matrix interactions) and affect both cell adhesion and viability [10, 23, 38]. Asami et al. [39] compared heat-treated ad non-heat-treated hydroxyapatite. They suggested that the promotion of osteoblast differentiation by the heat-treated material might have been caused by high concentration of the released calcium ions. These results were in agreement with research conducted by Orii et al. [4] who reported that, when osteoblasts were cultured in a medium containing a high level of calcium ions, the alkaline phosphatase activity increased rapidly when the formation of calcified materials began. In our study the results of ICP-OES measurements revealed a decrease in concentration of calcium in the case of all materials in comparison to the control (except from TC-2), particularly visible in the case of T-MgCHA. Materials containing calcite showed increased concentration of calcium ions, which may be connected with higher resorbability of calcite in comparison to the α-TCP and hydroxyapatite. Changes in calcium concentration in time may be connected to either release of Ca^2+^ ions from the material (increase of calcium concentration in SBF) or precipitation of calcium phosphates on the samples (decrease of calcium concentration in SBF). In our studies we demonstrated that the highest changes in concentration of ions (especially Ca^2+^) was noticed after 7 days, whereas most of in vitro studies on the cell lines is done between 24 and 72 h of incubation. It may suggest that future in vitro studies should be extended up to 7 days or even longer. In the case of material T-1 a considerable decrease in the concentration of magnesium was observed, however no negative impact on cell viability was noticed. A slightly lower number of cells treated with material T-AgHA extract is probably connected with the release of silver ions from Ag doped hydroxyapatite. In our studies 4 µg/g of silver was present in the T-AgHA samples. The amount of silver released from the material T-AgHA increased in time from 0.43 up to 1.31 mg/L what may influence the cellular response to a small extend. Similar effect, i.e., reduction in number of cells in comparison to the negative control, was observed by Pauksch et al. [40]. Pauksch noticed that the treatment of osteoblasts with 3 µg/g Ag_2_SO_4_ or AgNO_3_ salts (sources of silver ions) led to a severe drop in cell number compared to the negative control after 24 h. Furthermore in the case of silver nanoparticles—at higher concentrations AgNP-mediated cytotoxicity was observed. On the other hand, it is also well known that silver both ionic and metallic, as well as silver nanoparticles have an antibacterial effect and in vivo small amounts of Ag released from biomaterial may have a positive effect on healing process [41]. During in vitro tests some debris resulting from disintegration of bioceramics was visible. Although in the literature some negative impact of particulate debris on osteoblasts activity was reported [42], in our studies presence of CaPs debris did not have any negative impact on the cells.

## Conclusions

In our studies composites on the basis of highly reactive α-TCP powder combined with calcite or hydroxyapatite modified by Ag^+^, Mg^2+^, CO_3_^2−^ions were developed and tested. The influence of composition of the initial powder batches as well as the liquid phases (sodium alginate, chitosan and methylcellulose solutions) on cement setting process was confirmed. The addition of inorganic components to αTCP based powder batches increased both initial and final setting times. Moreover, it has been proven that calcite inhibits process of α-TCP hydrolysis to hydroxyapatite. The elongation of setting process was observed when chitosan solution in acetic acid was used whereas the presence of Na_2_HPO_4_ in the liquid phase resulted in decreasing of setting times. It was found that the parallel evaluation of ionic release and cytocompatibility of materials may be an effective tool for evaluation in vitro behavior of a material. In the case of our studies changes in the ionic culture environment, with respect to calcium, magnesium and phosphate ions did not have a negative influence on the activity of bone cells. All investigated composites can be considered as cytocompatible according to Part 5 ISO 10993 standard. Results of our research indicate that MgCHA, AgHA and calcite combined with highly reactive αTCP enhanced cell viability in comparison to only αTCP based material. A significant finding is also that materials containing chitosan and methylcellulose possessed higher cytocompatibility (~100% of control) than those with alginate (~80% of control). Moreover small amounts of CaPs ceramic debris resulting from disintegration of the samples did not have the negative impact on cells morphology or viability.

## References

[1] Bohner M, Galea L, Doebelin N (2012). Calcium phosphate bone graft substitutes: failures and hopes. J Eur Ceram Soc.

[2] Brown WE, Chow LC (1983). A new calcium phosphate setting cement. J Dent Res.

[3] Dorozhkin SV (2011). Medical application of calcium orthophosphate bioceramics. BIO.

[4] Orii H (1999). Effects of calcium ion on cell growth, mineralized nodule formation and gene expression of extracellular matrix proteins of osteoblast-like cells. Nihon Univ Dent J.

[5] Ormsby RW, Buchanan FJ, Best S, Cameron R, Dunne NJ (2014). Development of ion substituted calcium phosphate cement for spinal repair. Eur Cells Mater.

[6] Brunner TJ, Bohner M, Dora C, Gerber C, Stark WJ (2007). Comparison of amorphous TCP nanoparticles to micron-sized α-TCP as starting materials for calcium phosphate cements. J Biomed Mater Res B.

[7] Czechowska J, Zima A, Paszkiewicz Z, Lis J, Ślósarczyk A (2014). Physicochemical properties and biomimetic behavior of α-TCP-chitosan based materials. Ceram Int.

[8] Cicek G, Ayse Aksoy E, Durucan C, Hasirci N (2011). Alpha-tricalcium phosphate (a-TCP): solid state synthesis from different calcium precursors and the hydraulic reactivity. J Mater Sci.

[9] Brunner TJ, Grass RN, Bohner M, Stark WJ (2007). Effect of particle size, crystal phase and crystallinity on the reactivity of tricalcium phosphate cements for bone reconstruction. J Mater Chem.

[10] Przekora A, Czechowska J, Pijocha D, Ślósarczyk A, Ginalska G (2014). Do novel cement-type biomaterials reveal ion reactivity that affects cell viability in vitro?. Cent Eur J Biol.

[11] Vlad MD, Sindilar EV, Mariñoso ML, Poeata I, Torres R, López J, Barracó M, Fernández E (2010). Osteogenic biphasic calcium sulphate dihydrate/iron-modified α-tricalcium phosphate bone cement for spinal applications: in vivo study. Acta Biomater.

[12] Combes C, Bareille R, Rey C (2006). Calcium carbonate–calcium phosphate mixed cement compositions for bone reconstruction. J Biomed Mater Res Part A.

[13] Zima A, Paszkiewicz Z, Siek D, Czechowska J (2012). A Ślósarczyk, Study on the new bone cement based on calcium sulfate and Mg, CO_3_ doped hydroxyapatite. Ceram Int.

[14] Ewald A, Hösel D, Patel S, Grover LM, Barralet JE, Gbureck U (2011). Silver-doped calcium phosphate cements with antimicrobial activity. Acta Biomater.

[15] Lee GS, Park JH, Won JE, Shin US, Kim HW (2011). Alginate combined calcium phosphate cements: mechanical properties and in vitro rat bone marrow stromal cell responses. J Mater Sci.

[16] Kim IY, Seo S-J, Moon HS, Yoo MK, Park IY, Kim BC, Cho CS (2008). Chitosan and its derivatives for tissue engineering applications. Biotechnol Adv.

[17] Anselme K (2000). Osteoblast adhesion on biomaterials. Biomaterials.

[18] Coelho MJ, Fernandes MH (2000). Human bone cell cultures in biocompatibility testing. Part II: effect of ascorbic acid, beta-glycerophosphate and dexamethasone on osteoblastic differentiation. Biomaterials.

[19] Ascione F, Caserta S, Perris R, Guido S (2014). Investigation of cell dynamics in vitro by time lapse microscopy and image analysis. Chem Eng Trans.

[20] Buonomo R, Giacco F, Vasaturo A, Caserta S, Guido S, Pagliara V, Garbi C, Mansueto G, Cassese A, Perruolo G, Oriente F, Miele C, Beguinot F, Formisano P (2012). PED/PEA-15 controls fibroblast motility and wound closure by ERK1/2-dependent mechanisms. J Cell Physiol.

[21] Silano M, Vincentini O, Luciani A, Felli C, Caserta S, Esposito S, Villella VR, Pettoello-Mantovani M, Guido S, Maiuri L (2012). Early tissue transglutaminase-mediated response underlies K562(S)-cell gliadin-dependent agglutination. Pediatr Res.

[22] Liu YK (2009). The effect of extracellular calcium and inorganic phosphate on the growth and osteogenic differentiation of mesenchymal stem cells in vitro: implication for bone tissue engineering. Biomed Mater.

[23] Malafaya BP, Reis LR (2009). Bilayered chitosan-based scaffolds for osteochondral tissue engineering: Influence of hydroxyapatite on in vitro cytotoxicity and dynamic bioactivity studies in a specific double-chamber bioreactor. Acta Biomater.

[24] Z Paszkiewicz, A Slosarczyk, PL Patent No. 215450 B1 (2013)

[25] ASTM C266-04, Standard test method for time setting of hydraulic-cement paste by Gillmore needles, ASTM Annual Book of Standards, Pennsylvania 19428–2959.

[26] Washburn EW (1921). The dynamics of capillary flow. Phys Rev.

[27] 10993-12, ISO (2009). Biological evaluation of medical devices—Part 12: sample preparation and reference materials.

[28] 10993-5, ISO (2009). Biological evaluation of medical devices—Part 5: tests for in vitro cytotoxicity.

[29] Comley JC, Turner CH (1990). Potential of a soluble tetrazolium/formazan assay for the evaluation of filarial viability. Int J Parasitol.

[30] Ginebra MP, Boltong MG, Fernandez E, Planell JA, Driessens FCM (1995). Effect of various additives and temperature on some properties of an apatitic calcium phosphate cement. J Mater Sci.

[31] Momota Y, Miyamoto Y, Ishikawa K, Takechi M, Yuasa T, Tatehara S, Nagayama M (2004). Effects of neutral sodium hydrogen phosphate on the setting property and hemostatic ability of hydroxyapatite putty as a local hemostatic agent for bone. J Biomed Mater Res Part B.

[32] Espanol M, Perez RA, Montufar EB, Marichal C, Sacco A, Ginebra MP (2009). Intrinsic porosity of calcium phosphate cements and its significance for drug delivery and tissue engineering applications. Acta Biomater.

[33] Ginebra MP, Espanol M, Montufar EB, Perez RA, Mestres G (2010). New processing approaches in calcium phosphate cements and their applications in regenerative medicine. Acta Biomater.

[34] Boyan BD, Hummert TW, Dean DD, Schwartz Z (1996). Role of material surfaces in regulating bone and cartilage cell response. Biomaterials.

[35] Anselme K, Bigerelle M (2011). Role of materials surface topography on mammalian cell response. Int Mater Rev.

[36] Healy KE, Thomas CH, Rezania A, Kim JE, McKeownj PJ, Lom B, Hockberge PE (1996). Kinetics of bone cell organization and mineralization on materials with patterned surface chemistry. Biomaterials.

[37] Mohammadi M MShah, Chicatun F, Stähli C, Muja N, Bureau MN, Nazhat SN (2014). Osteoblastic differentiation under controlled bioactive ion release by silica and titania doped sodium-free calcium phosphate-based glass. Colloids Surf B.

[38] Cao N, Chen BX, Schreyer JD (2012). Influence of calcium ions on cell survival and proliferation in the context of an alginate hydrogel. Chem Eng.

[39] Asami A, Nakamura MI, Takeuchi M, Nakayama A, Nakamura H, Yoshida T, Nagasawa S, Hiraoka BY, Ito M, Udagawa NI, Miyazawa H (2008). Effects of heat treatment of hydroxyapatite on osteoblast differentiation. J Hard Tissue Biol.

[40] Pauksch L, Hartmann S, Rohnke M, Szalay G, Alt V, Schnettler R, Lips KS (2014). Biocompatibility of silver nanoparticles and silver ions in primary human mesenchymal stem cells and osteoblasts. Acta Biomater.

[41] Szczepanowicz K, Stefańska J, Socha RP, Warszyński P (2010). Preparation of silver nanoparticles via chemical reduction and their antimicrobial activity. Physicochem Probl Miner Process.

[42] Chiu R, Goodman SB. Biological response of osteoblasts and osteoprogenitors to orthopaedic wear debris, osteogenesis. In: Lin Y, editor. (2012). InTech, ISBN: 978-953-51-0030-0.

